# Di’ao Xinxuekang Capsule, a Chinese Medicinal Product, Decreases Serum Lipids Levels in High-Fat Diet-Fed ApoE^–/–^ Mice by Downregulating PCSK9

**DOI:** 10.3389/fphar.2018.01170

**Published:** 2018-11-01

**Authors:** Liping Qu, Didi Li, Xiaoping Gao, Yongwei Li, Jianming Wu, Wenjun Zou

**Affiliations:** ^1^School of Pharmacy, Chengdu University of Traditional Chinese Medicine, Chengdu, China; ^2^Department of New Drug Research and Development, National Engineering Research Center for Natural Medicines, Chengdu, China; ^3^Laboratory of Chinese Materia Medica, School of Pharmacy, Southwest Medical University, Luzhou, China

**Keywords:** Di’ao Xinxuekang, LDL-C, PCSK9, LDLR, ApoE^–/–^ mice

## Abstract

Numerous risk factors are responsible for the development of atherosclerosis, for which an increased serum level of low-density lipoprotein cholesterol (LDL-C) is a driving force. By binding to the low-density lipoprotein cholesterol receptor (LDLR) and inducing LDLR degradation, proprotein convertase subtilisin/kexin type 9 (PCSK9) plays a key role in cholesterol homeostasis regulation. The inducement of PCSK9 expression is also an important reason for statin intolerance. The Di’ao Xinxuekang (DXXK) capsule extracted from *Dioscorea nipponica* Makino is a well-known traditional Chinese herbal medicinal product used in atherosclerotic cardiovascular disease. Although DXXK has been widely used in atherosclerotic cardiovascular treatment for nearly 30 years, studies on the potential mechanisms of the lipid-lowering effect are very limited. The purpose of the present study was to demonstrate the possible involvement of the PCSK9/LDLR signaling pathway in the lipid-lowering and antiatherosclerotic effect of DXXK in high-fat diet-fed ApoE^–/–^ mice. The results showed that DXXK treatment alleviated hyperlipidemia, fat accumulation, and atherosclerosis formation in ApoE^–/–^ mice. Furthermore, changes in the expression of PCSK9 mRNA in liver tissue and the circulating PCSK9 level in ApoE^–/–^ mice were both reversed after DXXK treatment, and upregulation of LDLR in the liver was also detected in the protein level in DXXK-treated mice. Our study is the first to show that DXXK could alleviate lipid disorder and ameliorate atherosclerosis with downregulation of the PCSK9 in high-fat diet-fed ApoE^–/–^ mice, suggesting that DXXK may be a potential novel therapeutic treatment and may support statin action in the treatment of atherosclerosis.

## Introduction

Cardiovascular diseases (CADs) are the leading causes of death globally. Atherosclerosis, characterized by accumulation of macrophage and lipid and protein oxidation products in the intimal layer of arteries, is a fundamental pathological process of CADs (Wang et al., 2017). Although atherosclerosis involves a variety of risk factors in appropriate genetic backgrounds, hypercholesterolemia, defined by an overload of lipids as well as an imbalance of lipoproteins including low-density lipoprotein cholesterol (LDL-C) and high-density lipoprotein cholesterol (HDL-C), acts as a driving force and plays a vital role in the development and progression of atherosclerosis (Shin et al., 2014). A number of studies have indicated that both raising HDL-C and lowering LDL-C can result in a significant reduction in cardiovascular events due to an increased removal and reduced deposition of body cholesterol (Sirtori and Fumagalli, 2006; Park et al., 2018). Therefore, hypercholesterolemia and the following hyperlipidemia have come to be recognized as critical issues in the treatment of atherosclerosis.

The Di’ao Xinxuekang (DXXK) capsule, a traditional Chinese medicinal product extracted from the rhizome of *Dioscorea nipponica* Makino, has been used in the prevention and treatment of atherosclerotic CAD and other associated diseases for nearly 30 years in China (Yu et al., 2014). In 2012, this product was also approved in the Netherlands as the first traditional herbal medicinal product originating from outside of the EU area (Dutch Medicines Evaluation Board, 2012). Numerous studies have revealed that DXXK may increase blood flow and oxygen supply to the ischemic myocardium by vasodilatation (Chen et al., 1995), reduce consumption of myocardial oxygen via decreasing preload and afterload, maintain the activity of the Ca^2+^-ATP enzyme and Na^+^-K^+^-ATP enzyme by removing free radicals (Liu et al., 1994), and protect the cardiac cells from ischemia and reperfusion injury through preventing apoptosis and modulating the mitochondrial apoptotic pathway through attenuation of oxidative stress (Qin et al., 2014). In addition, several clinical reports have shown that DXXK can decrease the levels of total cholesterol, triglyceride (TG), and LDL-C and increase the serum HDL-C level as well as the APOA1/APOB ratio in patients with hyperlipidemia, which may be responsible for the effect of DXXK in alleviating atherosclerosis development (Zhou, 1997; Ji, 2001). However, studies on the mechanism of the potent lipid-lowering effect of DXXK are very limited. Our recent work demonstrated that the increase in HDL-C induced by DXXK may result from upregulating the synthesis of HDL by modulating the peroxisome proliferator-activated receptor γ (PPARγ)/liver X receptor α (LXRα)/ABCA1 pathway (Dong et al., 2017). However, the mechanism by which DXXK reduces the serum LDL-C level remains unclear.

Proprotein convertase subtilisin/kexin type 9 (PCSK9), belonging to the proprotein convertase family, plays a critical role in cholesterol homeostasis regulation by binding and degrading the low-density lipoprotein cholesterol receptor (LDLR), leading to a decrease in hepatic cholesterol uptake and an increase in circulating LDL-C (Horton et al., 2009). Loss-of- and gain-of-function PCSK9 variants have been detected in hypocholesterolemia and hypercholesterolemia patients, respectively (Zaid et al., 2008; Leigh et al., 2009; Eroğlu et al., 2018). Nonsense mutations in PCSK9 were relevant with the effect of lowering LDL-C and reducing cardiovascular events (Cohen et al., 2006). Therefore, since its discovery in 2003, PCSK9 has become a research hotspot in the development of new drugs to lower cholesterol and intervene in atherosclerosis (Reiss et al., 2018). Apolipoprotein E (ApoE), which is mainly synthesized in the liver and brain, is a glycoprotein that functions as a ligand for receptors that clear chylomicrons and very low-density lipoprotein (VLDL) remnants (Meir and Leitersdorf, 2004). The ApoE-knockout (ApoE^–/–^) mice can spontaneously develop hypercholesterolemia and atherosclerosis on a chow diet and have become a classic animal model for atherogenic hypercholesterolemia. Many studies have shown that profound interactions between diet and genetic factors influence atherogenesis (Reardon et al., 2003). Dietary factors, e.g., high fat, play a crucial role in the process and development of atherosclerosis (Kostogrys et al., 2012). A recent report from Zhao showed that a high-fat diet can increase PCSK9 expression in ApoE^–/–^ mice (Zhao et al., 2017). Therefore, in the present study, we detected the possible involvement of the PCSK9/LDLR signaling pathway in the antihyperlipidemic effects (more specifically, the LDL-C-lowering effect) of DXXK using high-fat diet-fed ApoE^–/–^ mice.

## Materials and Methods

### Materials and Reagents

The extract of the rhizome of *D. nipponica* Makino, which constitutes DXXK, was provided by the Chengdu Diao Pharmaceutical Group Co., Ltd. (Chengdu, China). High-performance liquid chromatography (HPLC) fingerprint analysis of the main chemical composition of DXXK was performed as we had previously reported (Yu et al., 2014) with the approved conditions for mobile phases. The mobile phases were utilized to elute the targets in a gradient mode (0–30 min: 15–35% A, 85–65% B; 30–75 min: 35–95% A, 65–5% B).

Colorimetric kits based on enzymatic reactions to determine mouse TC, TG, HDL-C, and LDL-C levels were purchased from Sangon Biotech (Shanghai, China). The TRIzol reagent was bought from Sigma-Aldrich Chemical (St Louis, MO). PrimeScript^TM^ RT Reagent Kit and SYBR Premix Ex Taq^TM^ were purchased from TaKaRa (Dalian, China). The LDLR antibody and mouse PCSK9 ELISA kit were purchased from Sino Biological (Beijing, China). The β-actin antibody, goat anti-rabbit IgG-horse radish peroxidase (HRP), and goat anti-mouse IgG-HRP were purchased from Santa Cruz Biotechnology (CA, United States). The bicinchoninic acid (BCA) protein assay kit was bought from Thermo Fisher Scientific (Mississauga, ON, Canada).

### Animals

Fifty 8-week-old male ApoE^–/–^ mice and 10 wild-type C57BL/6J mice weighing 18–22 g were purchased from Charles River Laboratories (Beijing, China). All animals were housed in standard cages (5 mice per cage) at a controlled ambient temperature of 25 ± 2°C and constant humidity of 60% with a 12-h light/12-h dark cycle. The experiment was approved by the Animal Ethics Committee of Chengdu University of Traditional Chinese Medicine, China. All procedures were performed according to the Guiding Principles for the Care and Use of Laboratory Animals of China.

### Experimental Design

All animals were given a normal chow diet for 1 week prior to the experiment. Following this acclimation, the 10 C57BL/6J mice used for the control group were fed a normal chow diet. The 50 ApoE^–/–^ mice were randomly divided into five groups (*n* = 10), the model group (A), 10 mg/kg atorvastatin group (B), 160 mg/kg DXXK group (C), 80 mg/kg DXXK group (D), and 40 mg/kg DXXK group (E) and fed a high-fat diet (0.25% cholesterol and 20% milk fat) for 18 weeks to induce the experimental model. In the meantime, mice in group B were orally administered with atorvastatin (10 mg/kg/d) while those in groups C, D, and E were given 160, 80, and 40 mg/kg DXXK orally for 18 weeks, respectively. Mice in group A received an equivalent volume of sterile water. All animals were fasted overnight and sacrificed at the end of the 18 weeks.

### Analyses of Serum Lipids

The blood samples were collected at the end of the 18-week experiment. The serum was collected by centrifugation of the blood samples at 3500 g (Sorvall ST-16R, Thermo) for 10 min at 4°C. The serum levels of total TC, TG, LDL-C, and HDL-C were measured through the use of the enzymatic colorimetric method with an automated biochemical analyzer using appropriate kits in accordance with the manufacturer’s instructions.

### Oil Red O Staining

Oil red O (Sigma, United States) staining was carried out as described previously (Rong et al., 2017). Briefly, after blood samples were harvested, the abdominal and thoracic cavities were opened via ventral incision. The liver tissues and aortas were dissected with external fatty deposits stripped under the dissection microscope. The aorta tissue included areas from the bifurcation of the aortic arch to the branching point of the right subclavian and common carotid artery. We subsequently opened the aorta laterally to expose the endothelial surface, stained the tissue with oil red O, and pinned the tissue on a wax surface. A BA200 digital microscopic camera system was utilized to capture the images. The percentage of the lipid accumulation area in the liver tissues and aortas stained by oil red O to the total luminal surface area was calculated through the use of the software program Image-Pro Plus 6.0 (Media Cybernetics, United States).

### Real-Time Reverse Transcription-Polymerase Chain Reaction (RT-PCR) Assays

Total RNA was extracted from the liver tissue by TRIzol reagent in accordance with the manufacturer’s instructions and isolated with chloroform and isopropanol. The total RNA concentration was determined by spectrophotometry. Afterward, cDNAs were synthesized from total RNA using the PrimeScript^TM^ RT Reagent Kit according to the manufacturer’s instructions. Hepatic mRNA expression of the genes was quantified by real-time RT-PCR using SYBR Premix Ex Taq^TM^. The primer sequences are shown in Table 1. The mRNA expressions of PCSK9 and LDLR were normalized by the corresponding β-actin. The PCR reaction was conducted according to the manufacturer’s instructions. The reaction solution included 10 μL of 2× diluted SYBR Premix Ex Taq II, 0.4 μM of the forward and reverse primer, and 2 μL of DNA template in a total 20 μL reaction. The reaction parameters were as follows: predenaturation at 95°C for 30 s, denaturation at 95°C for 30 s, annealing at 64°C for 20 s, and extension at 72°C for 20 s. After 40 cycles from denaturation to extension, the reaction solutions were incubated for an additional 60 s at 72°C and finally annealed at 55°C for 30 s. After amplification, standard curves were generated for target genes, which were then normalized against an endogenous reference gene β-actin. Relative expression of target genes was determined by the 2^-ΔΔCt^ method.

**Table 1:** The primer sequences for RT-PCR assay.

Gene	Forward primer	Reverse primer
LDLR	GACACCAAGGGCGTAA	TGGAATCAACCCAATAGA
PCSK9	GCCACAAGGACAGTCAA	AGGGCTCATAGCACATTA
β-actin	GGACTGTTACTGAGCTGCGTT	CAACCAACTGCTGTCGCCTT


### Enzyme-Linked Immunosorbent Assay (ELISA)

The level of serum PCSK9 protein was determined through the use of ELISA according to the manufacturer’s instructions. 50 μL of each serially diluted protein standard or serum sample and 150 μL of diluted dilution buffer were added to one well, which was incubated for 2 h at room temperature. After three washes with a washing buffer, a detection antibody was added and the solution was incubated for 2 h at room temperature. After three washes with a wash buffer, 200 μL of substrate solution was added, and the solution was incubated for another 20 min at room temperature, after which 50 μL of stop solution was added to detect the PCSK9 protein. Optical density values were read at 450 nm using an ELISA autoanalyzer (Perkin Elmer, United States). The concentrations of PCSK9 were determined in accordance with the standard curve diagram.

### Western Blot Analysis

Total proteins were extracted from liver tissue of mice. The liver tissue was homogenized in a radioimmunoprecipitation assay buffer containing protease inhibitors. Protein concentration was determined by BCA protein assay. Equal amounts of protein were electrophoresed on 10% density of sodium dodecyl sulfate–polyacrylamide gel electrophoresis gels, transferred to a polyvinylidene difluoride membrane, and blocked with 5% milk in phosphate buffered saline Tween 20 (PBST) for 2 h at 37°C. The proteins were then blotted with primary antibodies against LDLR (1:1000) or β-actin (1:800) overnight at 4°C and washed four times with 1× PBST. The blots were subsequently incubated for 1 h at 37°C with a 1:4000 dilution of goat anti-rabbit lgG-HRP or goat anti-mouse lgG-HRP and washed four times with 1 × PBST. The membranes were developed by incubation within the ECL Western blotting detection reagents. The β-actin was used as the control. ImageJ software was used to calculate the gray value of the band, which represented the protein expression level.

### Statistical Analyses

All data presented were expressed as the mean ± standard deviation (SD). Statistical differences between the groups were analyzed using one-way univariate analysis of variance (ANOVA). Differences at *P* < 0.05 were considered to be statistically significant (marked as ^∗^). The higher significance level was set at *P* < 0.01 (marked as ^∗∗^).

## Results

### HPLC Analysis of DXXK

**FIGURE 1:**
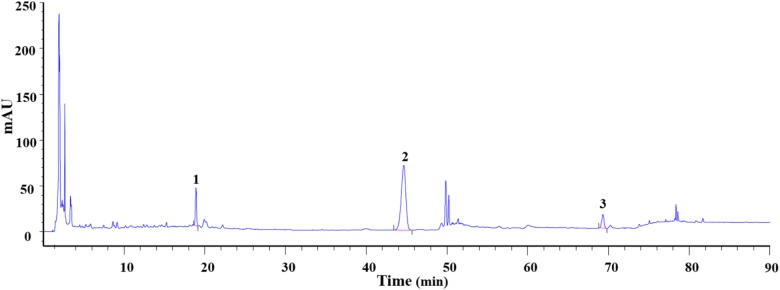
Analysis of protodioscin (1), pseudoprodioscin (2), and dioscin (3) concentrations in DXXK.

To determine the main chemical composition of DXXK, an HPLC fingerprint analysis was performed and the chromatogram is shown in Figure 1. The relative concentrations of protodioscin, pseudoprodioscin, and dioscin were 7.7, 25.4, and 4.4%, respectively. When compared with the DXXK drug standard in the Chinese Pharmacopoeia (2015 edition), the concentration of pseudoprodioscin is no less than 15% (Chinese Pharmacopoeia Commission, 2015).

### Effect of DXXK on Serum Lipid Levels

**FIGURE 2:**
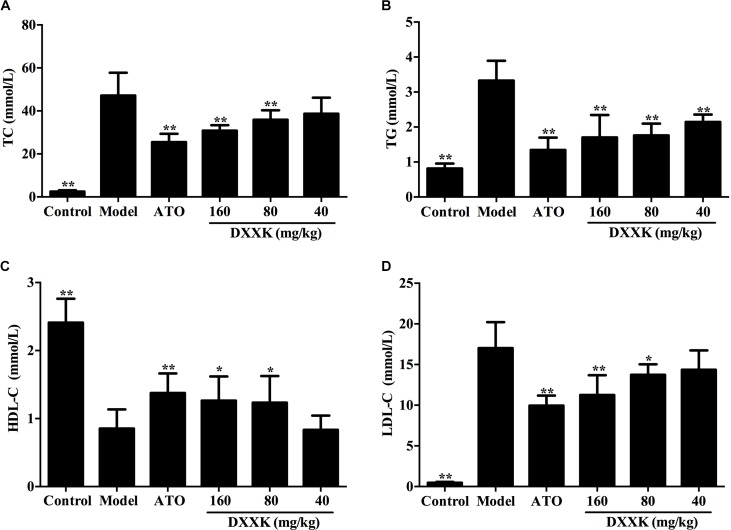
Effect of DXXK on serum lipid levels in ApoE^–/–^ mice fed a high fat diet. The DXXK treatment decreased the levels of serum TC **(A)**, TG **(B)**, and LDL-C **(D)** and increased the serum HDL-C **(C)**. ^∗^ Indicates a significant difference (*P* < 0.05) and ^∗∗^ (*P* < 0.01) versus model group. ATO, atorvastatin.

As a result of lipid metabolism disturbance, the model group mice displayed remarkable increases in the levels of serum TC, TG, and LDL-C and a decrease in the level of serum HDL-C compared with the control group (*P* < 0.01); data are shown in Figure 2. The DXXK (160, 80, and 40 mg/kg) and atorvastatin (10 mg/kg) treatment significantly decreased the levels of serum TC, TG, and LDL-C in high-fat diet-fed ApoE^–/–^ mice (*P* < 0.05 and *P* < 0.01). Moreover, compared with the model group, those given DXXK (160 and 80 mg/kg) and atorvastatin (10 mg/kg) also exhibited higher serum HDL-C levels.

### Effect of DXXK on Lipid Accumulation in the Aorta and Liver

**FIGURE 3:**
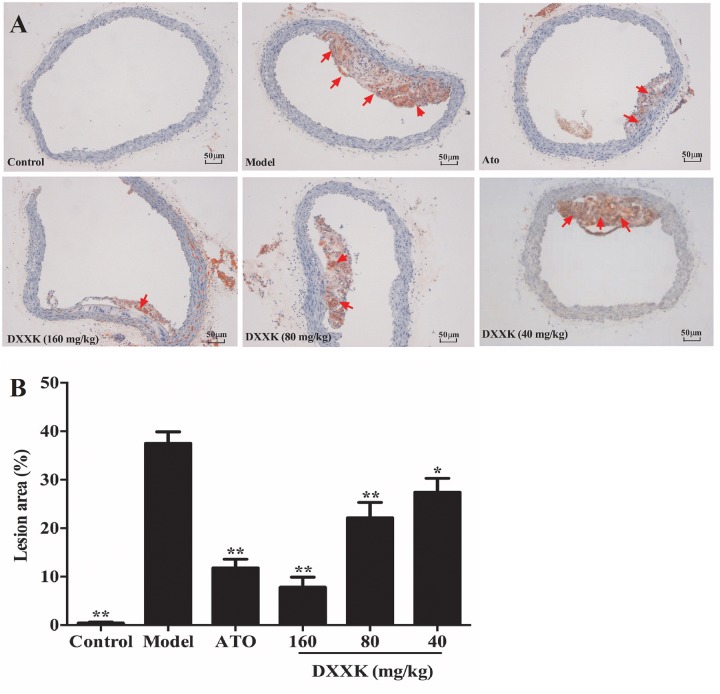
Effect of DXXK on the lipid accumulation in aortas of ApoE^–/–^ mice fed a high-fat diet. Representative photomicrographs Oil red O-stained fatty streaks in the aorta **(A)** (Magnification: ×400). Quantitative analysis of atherosclerotic lesions in the aorta section **(B)**. ^∗^ Indicates a significant difference (*P* < 0.05) and ^∗∗^ (*P* < 0.01) versus model group. ATO, atorvastatin.

As shown in Figure 3A, the atherosclerotic plaque area of the aorta cross-section in the high-fat diet-fed ApoE^–/–^ mice was significantly greater than that in the mice in the control group. A higher accumulation of lipid droplets with an area ratio of 36.3 ± 2.25% was presented in the aorta of the model group than in the control group mice. After treatment with atorvastatin (10 mg/kg) and DXXK (160, 80, and 40 mg/kg), the areas of plaques in the aorta decreased by different intensities (*P* < 0.05 and *P* < 0.01), with an area ratio of 10.65 ± 1.26, 7.603 ± 2.0, 22.9 ± 3.53, and 30.5 ± 3.9%, respectively (Figure 3B).

**FIGURE 4:**
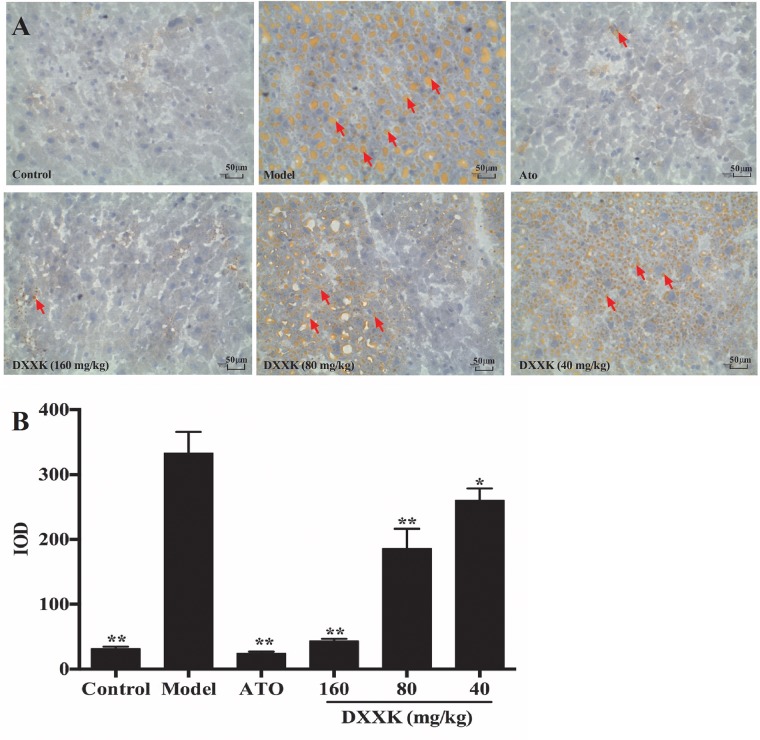
Effect of DXXK on the lipid accumulation in livers of ApoE^–/–^ mice fed a high-fat diet. Representative photomicrographs of oil red O-stained fatty streaks in the liver tissue **(A)** (Magnification: ×400). Quantitative analysis of atherosclerotic lesions in the liver section **(B)**. ^∗^ Indicates a significant difference (*P* < 0.05) and ^∗∗^ (*P* < 0.01) versus model group. ATO, atorvastatin.

Because the liver acts as a vital organ in the regulation of lipids, we also stained the liver tissue of mice to detect hepatic lipid accumulation with oil red O staining. As shown in Figure 4, more lipid droplets in liver tissues were detected in high-fat diet-fed ApoE^–/–^ mice than in the mice in the control group. Mice displayed decreases in lipid accumulation, both in the quantity and size of the lipid droplets, of different intensities after atorvastatin (10 mg/kg) and DXXK (160, 80, and 40 mg/kg) treatment (*P* < 0.05 and *P* < 0.01).

### Effect of DXXK on PCSK9 and LDLR mRNA Expression

**FIGURE 5:**
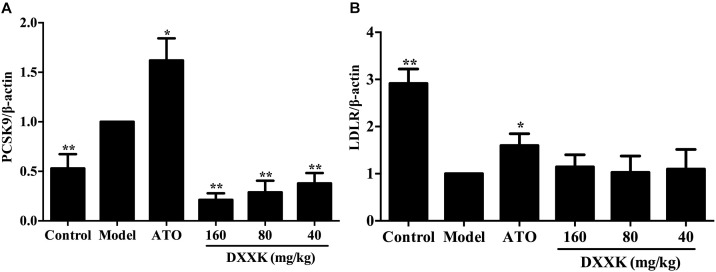
Effect of DXXK on the mRNA expression of PCSK9 **(A)** and LDLR **(B)** in ApoE^–/–^ mice fed a high-fat diet. ^∗^ Indicates a significant difference (*P* < 0.05) and ^∗∗^ (*P* < 0.01) versus model group. ATO, atorvastatin.

To examine the possible mechanism involved in the LDL-C-lowering effect of DXXK, levels of liver PCSK9 and LDLR mRNA were individually determined by real-time RT-PCR. As shown in Figure 5, compared with the expression in the control group, the expression of PCSK9 mRNA was increased and the expression of LDLR mRNA was decreased in high-fat diet-fed ApoE^–/–^ mice (*P* < 0.01). The DXXK (160, 80, and 40 mg/kg) treatment had a reversal effect on the expression of PCSK9 mRNA (*P* < 0.01), but had no effect on LDLR mRNA expression (*P* > 0.05). In contrast, atorvastatin (10 mg/kg) significantly increased PCSK9 and LDLR mRNA expression (*P* < 0.05).

### Effect of DXXK on the Serum PCSK9 Level and Protein Expression of Liver LDLR

**FIGURE 6:**
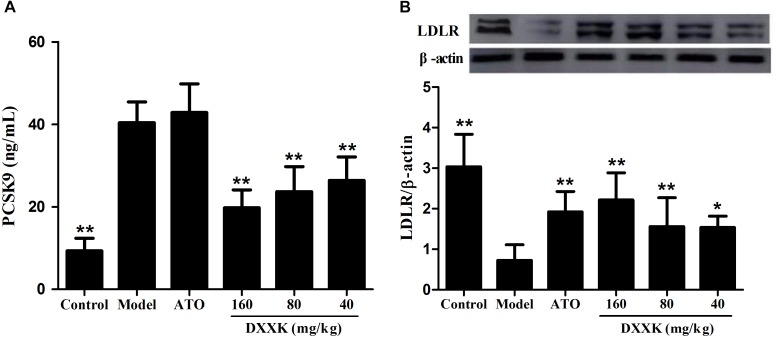
Effect of DXXK on serum PCSK9 level **(A)** and protein expression of liver LDLR **(B)** in ApoE^–/–^ mice fed a high-fat diet. ^∗^ Indicates a significant difference (*P* < 0.05) and ^∗∗^ (*P* < 0.01) versus model group. ATO, atorvastatin.

To confirm whether the LDL-C-lowering effect of DXXK involved the PCSK9/LDLR pathway, ELISA and Western blot analyses were performed to detect the level of serum PCSK9 and the protein expression of liver LDLR. As shown in Figure 6, ApoE^–/–^ mice fed a high-fat diet showed a significant increase in the level of serum PCSK9 and a remarkable decrease in the protein expression of liver LDLR compared with the mice in the control group (*P* < 0.01). The DXXK (160, 80, and 40 mg/kg) treatment had reversal effects both on the serum PCSK9 level and liver LDLR protein expression. However, atorvastatin (10 mg/kg) had no effect on the serum PCSK9 level (*P* > 0.05) in ApoE^–/–^ mice fed a high-fat diet, but significantly increased the protein expression of liver LDLR (*P* < 0.01).

## Discussion

Hypercholesterolemia is well known to be the critical step in the initial stage of the development of atherosclerosis and is strongly associated with CAD. Numerous previous studies have shown that the ability of statins to treat atherosclerosis is closely related to their antihyperlipidemic effects (Farnier and Davignon, 1998; Shin et al., 2014). In this present study, we observed the lipid-lowering and antiatherosclerotic effects of a traditional Chinese herbal medicinal product DXXK in ApoE^–/–^ mice fed a high-fat diet for 18 weeks. Our results demonstrated that the levels of serum TC, TG, and LDL-C were significantly decreased, while HDL-C was increased after DXXK treatment. These results are consistent with previous clinical reports (Zhou, 1997) in China. The oil red O staining results also showed that DXXK treatment inhibited lipid accumulation in the liver tissue and diminished arteriosclerosis plaque formation in ApoE^–/–^ mice.

Hepatic uptake, mediated via transmembrane LDLR that internalizes bound LDL particles via endocytosis, is the major way in which circulating LDL-C is removed from the plasma (Urban et al., 2013). The PCSK9, an enzyme produced mainly in the liver, plays a critical role in cholesterol homeostasis regulation by binding LDLR and inducing LDLR degradation (Horton et al., 2009). Thus, we further examined the possible involvement of the PCSK9 signaling pathway in the LDL-C-lowering effect of DXXK. The PCSK9 is expressed from a single gene. The most important feature of its synthesis involves its autocleaving and autocatalytic processing in the endoplasmic reticulum, which results in mature PCSK9 capable of binding LDLR (Dixon et al., 2016). Mature PCSK9 is subsequently secreted into circulation from hepatocytes and functions by binding to the epidermal growth factor-like repeat A (EGF-A) domain on LDLR, resulting in hepatocyte endocytosis and lysosomal degradation (Dixon et al., 2016). A previous study demonstrated that PCSK9 mostly acts extracellularly to cause subsequent degradation of LDLRs in liver cells; moreover, the study suggested that if an intracellular pathway exists, it only plays a minor role in LDLR regulation (McNutt et al., 2009). Our results indicated that the changes in the expression of PCSK9 mRNA in the liver and circulating PCSK9 levels were both reversed and downregulated after DXXK treatment in high-fat diet-fed ApoE^–/–^ mice. Furthermore, upregulation of LDLR in the liver was also detected at the protein level in DXXK-treated mice. Therefore, our research suggested that the possible mechanism by which DXXK lowers LDL-C levels and ameliorates atherosclerosis is via inhibiting the PCSK9/LDLR signaling pathway.

Statins are the inhibitors of the limiting enzyme named 3-hydroxy-3-methylglutaryl-coenzyme A reductase (HMG-CoA) in the cholesterol biosynthetic pathway and have been used as the first-line treatment for hypercholesterolemic patients for more than two decades. However, a significant number of high-risk patients still fail to achieve LDL-C targets, which is directly related to an increased risk of cardiovascular events (Garattini and Padula, 2017). Statins achieve their LDL-C-lowering effects via inhibiting HMG-CoA to modulate sterol regulatory element-binding protein-2 (SREBP-2), which upregulates the hepatic expression of LDLR, resulting in enhanced LDL-C clearance from circulation (Careskey et al., 2008). Unfortunately, PCSK9 expression is also regulated by SREBP-2 and can be induced by statins both at moderate and high doses (Careskey et al., 2008; Welder et al., 2010). Studies have also shown that when statins induce expression of PCSK9 to a greater extent than the expression of LDLR, there is an increase in serum LDL-C, causing resistance to the LDL-cholesterol-lowering effect of statins and resulting in statin intolerance (Dong et al., 2010). In the present study, we demonstrated the inhibitory role of DXXK in the PCSK9/LDLR signaling pathway in ApoE^–/–^ mice, while confirming that atorvastatin (10 mg/kg) promoted the upregulation of PCSK9 as well as LDLR. Moreover, methyl protodioscin, a molecule similar to the key component protodioscin in DXXK, has also been indicated to be able to reduce LDL-C via regulating SREBP, intervening in downstream gene targets including PCSK9, and protodioscin was shown to further promote LDLR expression through reducing the PCSK9 level *in vitro* (Ma et al., 2015). Currently available data have demonstrated fewer untoward muscle-related adverse effects as well as greater LDL-C reductions in statin-intolerant patients administered with the fully monoclonal antibody to PCSK9 (Moriarty et al., 2015; Nissen et al., 2016). Findings from a meta-analysis of randomized controlled trials have indicated that evolocumab could be a hopeful agent for challenging patients, such as those having statin intolerance or patients who fail to attain the target goal of LDL-C despite consumption of maximum doses of statins (Eslami et al., 2017). Our results suggest that DXXK, a marketed product commonly used in atherosclerotic CAD, has good clinical value and may support statin action due to the reversal of PCSK9/LDLR expression in ApoE^–/–^ mice revealed in our study.

**FIGURE 7:**
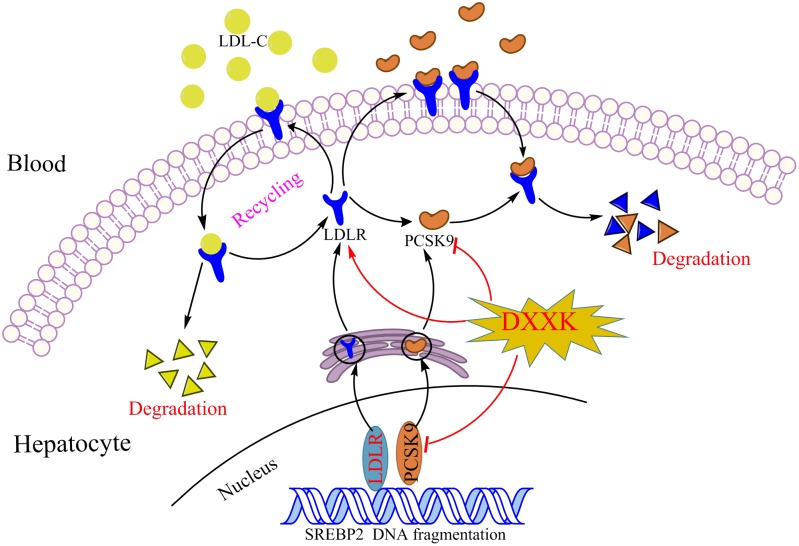
The DXXK decreases serum LDL-C level through PCSK9/LDLR pathway.

In summary, DXXK may simultaneously reduce the levels of three major modifiable lipid risk factors, LDL-C, HDL-C, and TG, and we revealed that the possible mechanism by which DXXK lowers LDL-C levels and ameliorates atherosclerosis involves its inhibitory role in the PCSK9/LDLR signaling pathway (Figure 7). The mechanism involved in the degradation of LDLR by PCSK9 is extremely complex and has only begun to be understood. However, regardless of the exact mechanism, a decrease in the LDLR level results in a decreased ability of the liver to bind LDL from circulation. Therefore, further exploration of the regulatory effect of DXXK on the affinity of PCSK-9 to bind with the EGF-A of LDLR might be worthwhile. In addition, the E3 ubiquitin ligase Mylip/Idol has been reported to be another factor that may regulate the LDLR level as well as the uptake of lipoproteins into the liver and other tissues (Zelcer et al., 2009; Hong et al., 2010; Do et al., 2012). Whether DXXK can affect the E3 ubiquitin ligase Mylip/Idol is another promising point to determine the mechanisms by which DXXK degrades LDLR.

## Author Contributions

LQ designed and wrote this manuscript. DL conducted the research and analyzed the data. YL contributed to the HPLC fingerprint analysis. XG contributed to the oil red O staining analysis and revised the manuscript. JW and WZ designed and funded the research, interpreted the data, and finally approved the submission of this manuscript. All authors have read and agreed with the manuscript.

## Conflict of Interest Statement

The authors declare that the research was conducted in the absence of any commercial or financial relationships that could be construed as a potential conflict of interest.
